# Biomimetic Strategies for Developing Abiotic Stress-Tolerant Tomato Cultivars: An Overview

**DOI:** 10.3390/plants12010086

**Published:** 2022-12-23

**Authors:** Gyanendra Kumar Rai, Pradeep Kumar, Sadiya Maryam Choudhary, Rafia Kosser, Danish Mushtaq Khanday, Shallu Choudhary, Bupesh Kumar, Isha Magotra, Ranjit Ranjan Kumar, Chet Ram, Youssef Rouphael, Giandomenico Corrado, Tusar Kanti Behera

**Affiliations:** 1 School of Biotechnology, Sher-e-Kashmir University of Agricultural Sciences and Technology of Jammu, Jammu 180009, India; 2 Division of Integrated Farming System, ICAR—Central Arid Zone Research Institute, Jodhpur 342003, India; 3 Division of Plant Breeding and Genetics, Sher-e-Kashmir University of Agricultural Sciences and Technology of Jammu, Jammu 180009, India; 4 Division of Entomology, Sher-e-Kashmir University of Agricultural Sciences and Technology of Jammu, Jammu 180009, India; 5 Division of Biochemistry, ICAR—Indian Agricultural Research Institute, New Delhi 110001, India; 6 Division of Crop Improvement, ICAR—Central Institute for Arid Horticulture, Bikaner 334006, India; 7 Department of Agricultural Sciences, University of Naples Federico II, Portici, 80055 Naples, Italy; 8 ICAR—Indian Institute of Vegetable Research, Jakhini (Shahanshapur), Varanasi 221305, India

**Keywords:** *Solanum lycopersicum*, biotechnology, drought, salinity, improvement

## Abstract

The tomato is one of the most important vegetables in the world. The demand for tomatoes is high in virtually any country, owing to their gastronomic versatility and nutritional and aromatic value. Drought, salinity, and inadequate temperature can be major factors in diminishing yield, affecting physiological and biochemical processes and altering various metabolic pathways, from the aggregation of low molecular–weight substances to the transcription of specific genes. Various biotechnological tools can be used to alter the tomato genes so that this species can more rapidly or better adapt to abiotic stress. These approaches range from the introgression of genes coding for specific enzymes for mitigating a prevailing stress to genetic modifications that alter specific metabolic pathways to help tomato perceive environmental cues and/or withstand adverse conditions. In recent years, environmental and social concerns and the high complexity of the plant response may increase the attention of applied plant biotechnology toward biomimetic strategies, generally defined as all the approaches that seek to develop more sustainable and acceptable strategies by imitating nature’s time-tested solutions. In this review, we provide an overview of some of the genetic sequences and molecules that were the objects of biotechnological intervention in tomato as examples of approaches to achieve tolerance to abiotic factors, improving existing nature-based mechanisms and solutions (biomimetic biotechnological approaches (BBA)). Finally, we discuss implications and perspectives within the GMO debate, proposing that crops modified with BBA should receive less stringent regulation.

## 1. Introduction

Despite yearly fluctuations, the tomato (*Solanum lycopersicum* L.) remains a widely grown vegetable and has significant nutritional and economic importance all over the world [1]. For example, the lycopene in tomato fruits provides antioxidative and anticancerous properties. Moreover, its versatility and vivid color make the tomato a favorite food in many countries. In 2020, the area under tomato production was approximately 5.05 Mha, with a fresh production of 186.8 Mtones [2]. The demand for tomatoes on a global scale has increased in the last century because of their wide range of uses as raw, cooked, and processed foods. 

Tomato yield, like many other vegetables, is limited by abiotic stress, such as cold, heat, drought, and salinity [3,4]. The main environmental issues reducing tomato cultivation are low-quality water, extreme temperature, an imbalance in the nutritional content of the soil substrate, elemental toxicity, and high salinity [3,5,6]. Abiotic stress becomes highly problematic in the open field, where stresses can easily coexist. Improving the tomato’s ability to withstand common abiotic stresses is economically more advantageous and sustainable than the use of non-renewable chemical input or the implementation of new agronomic measures. It is, therefore, necessary to develop resilient, high-yielding cultivars with greater tolerance to a variety of abiotic challenges [7,8]. Classic tomato breeding has increased yield (and its specific components), quality-related traits, and resistance to some abiotic as well as biotic stress considerably. The application of high throughput and multidisciplinary methodologies currently provides new tools to develop stress tolerance for crop species with high genetic complexity and large ecological interactions like tomato [9].

It has long been established that recombinant DNA technology is a suitable approach for breeding tomato cultivars. Developments in tomato genomics have largely favored the identification of various genes, gene families, and metabolic pathways that are responsible for providing the required modified adaptation to the plants to stressful environments. Their study and modification can result in improved yield attributes under sub-optimal environmental conditions [10,11,12]. In addition to being the foundation of novel cultivars, genetically modified plants can also be useful for studying and characterizing how gene networks for abiotic stress tolerance interact and perform under a variety of conditions [13]. In this article, we present and discuss various genes, proteins, and other molecular compounds that are directly and/or indirectly linked to improved tolerance to abiotic stress, focusing on biomimetic strategies. Broadly speaking, a biomimetic technological approach is one that follows and improves models provided by nature. Although it is debated whether “biomimetics” should be distinguished from “biomimicry” (interventions that are only inspired by vs. those strictly following nature), we discuss the principles of the mechanisms of stress response in tomato, as well as various possible interventions, under the conceptual framework that the study of nature’s models provides inspiration to their modifications and improvements. To address public concerns, the concepts of cisgenesis and intragenesis have been developed as a more acceptable strategy to alter the genetic material of a plant. Nonetheless, it is not easy to strictly define the extent of the sequence variability of a gene or, more generally, a DNA stretch that can be considered within or outside the gene pool of a crop species. This is further complicated considering that cisgenesis and intragenesis allow the use of sequences from not only the crop of interest but also related species capable of sexual hybridization. To stimulate a discussion, in this review, we would like to put forward that modifications of the metabolism of an organism through DNA recombinant technology should be evaluated considering not only the sequence of interest but also the functional modification that will be achieved. To this purpose, we refer to the term biomimetic biotechnological approaches (BBA) to identify the improvement, but not the redesign or (non-native) reconstruction, of systems and pathways that are already present within a plant species and active in an organ or tissue, irrespective of the taxonomical source of the DNA employed. Biomimetics is often coupled with aterials science, mechanical engineering, and nanotechnology and, along with biomimicry, is associated with more biodiverse agricultural systems. Therefore, the concept of BBA in plants may facilitate the dialogue as well as the cultural exchange between scientists, stakeholders, and citizens.

## 2. Abiotic Stress Resistance at the Physiological Level

**Figure 1:**
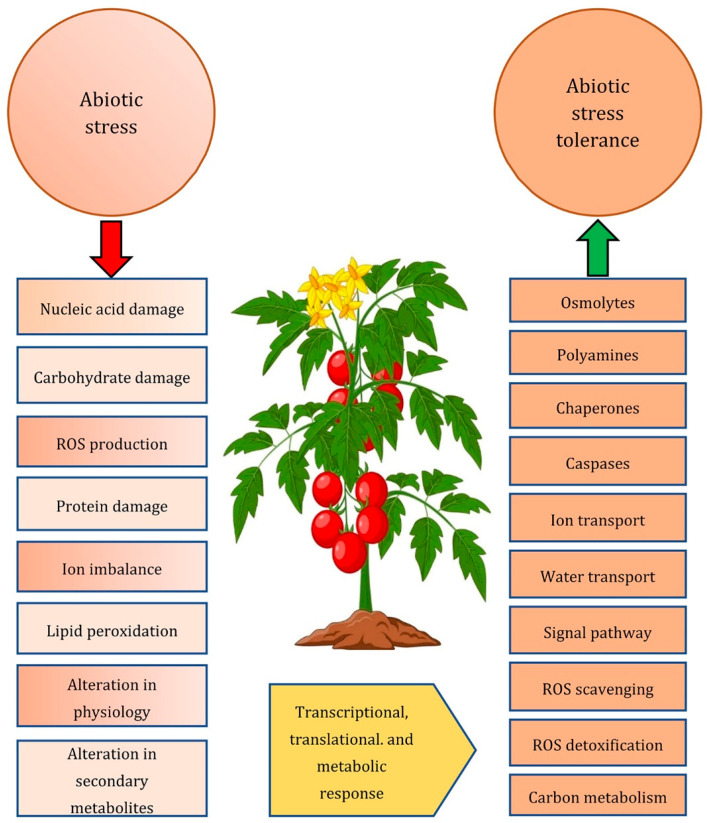
A schematic illustration of the effects of abiotic stress at the cellular level. The tolerance of tomato in a challenging environment requires several modifications to its cellular activities, which are based on stress-induced gene expression, translational reprogramming, and metabolic adaptation, often based on homeostatic mechanisms.

Abiotic stress causes a range of changes in morphological, physio-biochemical, and molecular processes in plants [14]. Extreme heat, salt, oxidative stress, and drought are frequently linked and can cause cellular damage either alone or in a combined fashion. The abiotic stresses provide extremely complex stimuli with a wide range of concurrent but distinct characteristics. Each of them has the potential to provide the plant cell with a totally distinct set of environmental cues [15]. On the whole, abiotic stress leads to changes in osmotic balance and ion distribution, which in turn affect both individual cells and the entire plant. Ion and water homeostasis change, causing cellular disturbance, growth arrest, and even death in the most severe cases [16]. Although plant response is one multifaceted and holistic adjustment to the environment, three interrelated activities can be distinguished as crucial for abiotic stress tolerance. The first activity is to stop or lessen the damage, the second one is to restore homeostatic conditions in the new challenging environment, and the third is to restore plant growth (i.e., recovery stage) [17]. Resistance or tolerance must, therefore, assume some flexibility in the metabolic processes that enable plants to survive in harsh conditions (Figure 1). The development in the understanding of plant response to abiotic stress at various levels helps explain these complicated cellular reactions. On a time scale, during abiotic stress, in theory, three phases can be discerned: (1) the phase of alarm; (2) the period of defense; and (3) the phase of collapse [18]. However, an alternative stage should also be considered, the regeneration stage [19]. This is highly crucial and allows the retrieval of various physiological functions of the plant [20]. 

**Figure 2:**
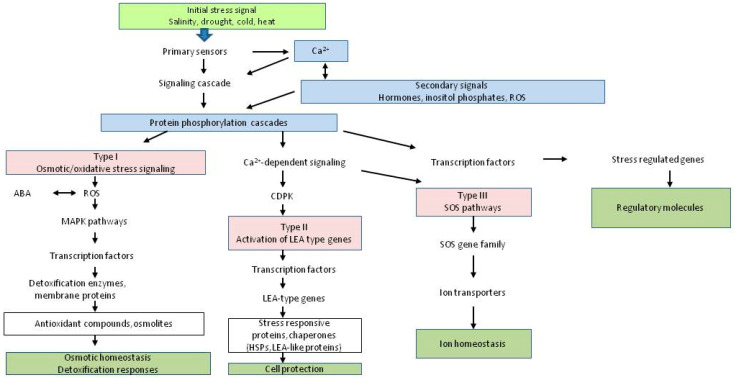
Abiotic stress at the physiological level is mediated by the signal transduction pathway. The first stage of a signal transduction pathway is signal perception, which is followed by the formation of secondary messengers (e.g., calcium, phosphoinositides, and reactive oxygen species (ROS)). Second, messengers promote the downstream signaling cascades that lead to the plant response against abiotic stress.

The stress response is integrated, incorporating several pathways, certain cellular sections, and tissues, and it relies on a battery of signaling molecules to synchronize the response of a specific variation at the organismal level. In response to abiotic stresses, various pathways are activated concurrently (Figure 2). Molecular and genetic investigations have shown that these pathways are highly complex, owing to the diversity of information involved. As plants respond to challenges physiologically, molecularly, and cellularly, it is not a surprise that the transcription of several dissimilar resistance/tolerance genes is also altered [21].

## 3. Biotechnological Tools to Develop Tolerant Plants against Abiotic Stress

Biotechnological tools include several methods used for plants to develop tolerance to abiotic stress. The genetic transformation of tomato relies highly on the tissue culture technique [22]. Recent advances in the field of plant genetic transformation have enabled the identification of genes that are responsible for tolerance to different environmental stresses [23,24]. Further advancement in understanding the genomics of wild relatives of tomatoes and other Solanaceae has facilitated their exploitation in various breeding programs aiming to introgress genes responsible for abiotic stress resistance in cultivars.

### 3.1. Genetic Transformation Methods in Tomato 

Multiple transformation techniques have been utilized to deliver foreign DNA sequences into an ample range of plant species [25]. The combination of recombinant DNA technologies, genetic transformation, and plant tissue culture are at the core of the production of transgenic plants in a variety of crops [26,27,28,29,30,31,32,33,34,35,36]. In tomato, the first genetic transformation protocol was developed in the 1980s [37], and still today, *Agrobacterium*-mediated techniques are widely employed for many tomato cultivars [38]. Transformation mediated by the *Agrobacterium* is a complex process. Briefly, the efficiency of gene delivery into tomato plants depends on various factors, such as the pre-culture of the explants, culture media, culture density, virulence and strain of *Agrobacterium*, phytohormones, type of explants, vectors, size of DNA insert, and genotype of the recipient plant [39,40]. Table 1 presents a short selection of research efforts devoted to improving the process of tomato genetic transformation. Genetic transformation can also be obtained using *A. rizogenes* [41]. However, some detrimental phenotypes can be observed in tomato plants, such as shortened internodes, reduced seed setting, and wrinkled leaves. *A. rizogenes*–mediated transformation can be utilized for the in vitro production of compounds in tomato with biopharmaceutical properties. Besides indirect genetic transformations, direct methods like particle bombardment have also been reported for tomato [42]. This method was optimized by altering factors such as the quality and quantity of DNA, concentration of osmoticum in the tissue culture media, firing separation, and period of particle bombardment to which tomato explants are exposed [43]. 

Besides the addition of a new DNA sequence or (untargeted) mutation of the tomato genome, recent advances in recombinant DNA technology and reverse genetic approaches, such as antisense technology, RNA interference (RNAi), and genome editing by CRISPR-CAS9, have revolutionized functional genomics in plants. These approaches have been utilized in tomato cultivars to delay their ripening during abiotic stress, such as extreme temperature, by silencing the gene *vis 1* [54,55]. 

Overall, the genetic transformation of tomato is a mature and well-established technique that is employed by numerous laboratories around the world. Although improvements in regeneration and transformation efficiency are always welcome, the production of genetically modified tomatoes should not be considered a limiting factor for biotechnological approaches since efficient and repeatable transformation and regeneration protocols are widely available.

### 3.2. Transformation Approaches Using rDNA Technologies (Genetic Engineering)

Climate change is predicted to increase the occurrence of abiotic stress, further hampering the ability of plants to yield [56]. Traditional plant breeding has limitations for creating a substantial level of tolerance against abiotic stress because it is time-consuming and often requires a complex breeding scheme to insert multiple sources of variability from wild relatives to a cultivated variety. Recombinant DNA (rDNA) technology–based tools have been traditionally considered alternatives to change the genetic constitution of plants. Different rDNA technologies have been employed to modify the tomato genome so that it can adapt to abiotic stress. These modifications include the exploitation of regulatory genes highly expressed during stress and coding for enzymes whose biochemical or enzymatic activity is useful to counteract abiotic stress [57]. Various examples are reported in Table 2. 

Genetic modifications based on sequences that encode compounds involved in stress adaptation are given below.

#### 3.2.1. Mannitol

Mannitol is an important polyol (sugar alcohol) produced from fructose metabolism and serves as a scavenger of free radicals and osmoregulation. The enzyme involved in fructose metabolism to obtain mannitol is mannitol-1-phosphate dehydrogenase, and the corresponding gene encoding this enzyme is *mt1D* [89,90]. In tomato, the constitutive expression of a bacterial *mt1D* gene driven by the CaMV 35S promoter provides improved tolerance against chilling, drought, and saline stress [72]. 

#### 3.2.2. Glycine Betaine

It is an organic compound derived from the amino acid glycine, whose accumulation in plants may occur following abiotic stress. In plants, this compound is considered an organic osmolyte, ensuring, for instance, the regulation and preservation of the thylakoid membrane and, thus, sustaining the photosynthetic efficiency under stress [91]. Various studies have used biotechnological tools, such as the overexpressing of this compound, to facilitate an increased response of plants to abiotic stress tolerance. For example, the expression of the bacterial choline oxidase A (coda) in tomato targeted to the chloroplasts with a transit peptide resulted in an accumulation of glycine betaine in a relatively low (0.09 to 0.30 µmol·g^−1^ FW) but significant (up to 86% in chloroplasts compared to unstressed control plants) amount, sufficient to enhance tolerance to chilling at various phenological stages, as indicated by an increased yield in stress conditions of the transgenic plants [88]. 

#### 3.2.3. Glutathione

Glutathione, an important antioxidant performing multiple functions in plants, is synthesized from amino acids (i.e., L-glutamate, cysteine, and glycine). The whole process requires two ATP molecules and is catalyzed by two glutamate enzymes—cysteine ligase (GCL) and glutathione synthetase (GSS). This tripeptide provides protection at cellular and tissue levels in response to various reactive oxygen species (ROS), such as peroxides, superoxides, and hydroxyl radicals [92]. Glutathione has an important role during induced stress. The constitutive expression in tomato of a Se-independent glutathione peroxidase (GPx5) from *Mus musculus* resulted in an increased tolerance to mechanical stress [93]. Similarly, the concurrent constitutive expression of two glyoxalase (GlyI and GlyII) from *Brassica juncea* in tomato showed a reduced growth depression and membrane damage (as indicated by the level of lipid peroxidation and hydrogen peroxide production in leaves) following long-term exposure (3 months) to salinity (up to 800 mM NaCl) [94].

#### 3.2.4. Osmotin

Osmotin is a 26 kDa protein, a member of the PR-5 family, which also includes zeamatin and thaumatin. It accumulates in plants as a defense mechanism against abiotic stress because of its prominent role in osmoregulation [95]. It has been reported that tomatoes constitutively expressing an osmotin gene from *Nicotiana tabacum* have higher levels of proline, increased chlorophyll contents, and higher water contents (under stress). These features were considered crucial in helping tomato withstand salt stress (150 mM NaCl for 10 days) [87]. The osmotin from *N. tabacum* was also used to increase pathogen resistance in transgenic barley, while it did not have a significant impact on insect-borne virus infections (by aphids and leafhoppers) [96]

#### 3.2.5. Polyamines

Polyamine (PA) is a term used to indicate the wide class of organic molecules having multiple (more than two) amino acid groups. In plants, naturally occurring, low molecular–weight polyamines are associated with embryogenesis, organogenesis, anthesis, fruit development, ripening, and leaf senescence, but there is also evidence of their role in stress response [95]. The most common and abundant PAs in plants are putrescine (a diamine) and its derivatives spermidine and spermine [97]. Tomato transformed to constitutively express the arginine decarboxylase gene from *Poncirus trifoliata* (PtADC), indirectly involved in the biosynthesis of putrescine, showed increased levels of free PAs and improved tolerance to leaf dehydration and drought stress [98]. Tomato genetically transformed to constitutively overexpress the tomato *SlSAMS_1_* gene accumulated PAs and hydrogen peroxide and had an improved alkali stress tolerance. This gene is a member of the S-adenosylmethionine synthetase (SAMS) family, and it is stress-inducible. These genes catalyze the formation of SAM, which is also a precursor to PAs [99]. 

#### 3.2.6. Trehalose

Trehalose is a highly soluble disaccharide made of glucose subunits, and it is present in a wide range of organisms, including prokaryotes, algae, mosses, fungi, protozoa, and mammals. Trehalose appears to be able to play a special function as a stress metabolite protecting the integrity of the cell against environmental stress and nutrient limitations [100]. Traditionally, trehalose has been of little importance for angisoperms, where another non-reducing saccharide, sucrose, has a predominant role in carbon storage and transport. Nonetheless, the discovery of gene families encoding trehalose phosphate synthases (TPSs) and trehalose phosphatases, along with their subsequent functional characterization, indicated that trehalose acts mainly as an osmoprotectant and as a signal molecule involved in stress response. Recombinant DNA technologies have made it possible to modify genes governing trehalose metabolism in tomato. For example, the constitutive expression of *Saccaromyces cerevisiae ScTPS1* improved tolerance against drought or salt. Nonetheless, transgenic plants had phenotypic abnormalities and alterations in carbohydrate biosynthesis [101].

#### 3.2.7. Biosynthesis of Ethylene 

Ethylene is a well-known plant hormone whose commercial derivatives are also used to induce post-harvest tomato ripening. Because of its applied importance, there are several studies on genetically modified tomato cultivars with altered ethylene pathways in relation to fruit maturation. Ethylene production is typically increased in stressful environmental conditions. Several works have demonstrated that one of the positive effects of plant growth–promoting rhizobacteria (PGPR) is lowering the ethylene level under stress by cleaving and deaminating aminocyclopropane-1-carboxylic acid (ACC), the precursor of ethylene, by ACC-deaminases [102]. Tomato cultivars expressing a bacterial ACC deaminase under constitutive and inducible promoters were more tolerant to flooding [103].

#### 3.2.8. Aquaporins

Aquaporins (AQPs) are trans-membrane proteins that allow the movement of water and small solutes between and within cells. Numerous studies have reported the potential roles of aquaporins in relation to abiotic stress in plants, water use efficiency (WUE), and solute transport in plants [104,105]. In tomato, over forty members of the AQP gene family have been linked to abiotic stress and plant development, mainly because of their expression pattern [106]. The overexpression of genes regulating the formation and functioning of aquaporins, such as *SlTIP;2*, in tomato increased the tolerance to abiotic stress. Interestingly, transgenic plants were more productive and had higher biomass than untransformed controls in normal and drought conditions [107]. An AQP from apple (MdPIP1;3) was also used to increase fruit growth rate and size mainly thanks to bigger cells, also increasing tolerance to drought stress [86]. Similarly, the overexpression of *SlPIP2;1* conferred to tomato’s higher hydraulic conductivity and tolerance against drought stress [108,109].

#### 3.2.9. Heat Shock Proteins

A set of relatively conserved, ubiquitous proteins, referred to as heat shock proteins, are synthesized by virtually all organisms, including plants, in response to various environmental stresses. These proteins often serve as intracellular chaperones and, for the establishment of protein-protein interaction, are involved in protein folding, assembly, translocation, degradation, and transport [110]. Different genes encoding HSPs (e.g., *HsfA1*, *HsfA2*, *HsfB1*, *LeHSP 17.6*) have been identified and delivered to tomato to facilitate the production of HSPs, with the common aim of helping plants better adapt to stress [111,112]. Moreover, the overexpression in tomato of *LeHSP21.5* diminished tunicamycin-induced ER stress [85]. Tunicamycin is an antibiotic that inhibits protein N-glycosylation, hence, inducing misfolded glycoproteins, and it is experimentally used to induce the unfolded protein response in living organisms. Furthermore, other plant chaperonins have been employed to increase the stress resistance in tomato [113]. For example, the overexpression of the tomato *SlDnaJ20* relieved ROS accumulation by ensuring high levels of SOD and APX activities and was associated with higher fresh weights of six-week-old plants under heat stress [114].

#### 3.2.10. Antioxidants

Many antioxidants have been reported in plants that act as buffers to regulate the redox potential of cells. Among antioxidant enzymes, the most exploited in plant biotechnology are probably glutaredoxins, catalases, ascorbate peroxidases (APX), and superoxide dismutases (SOD). Just to give a few examples of applications, a catalase gene (*katE*) from *E. coli*, introduced in the chloroplast genome of tomato under the RBCS promoter, increased catalase activity and better protected plants from oxidative stress induced by high light intensity, drought, or low temperature, compared to the untransformed control [76]. An ascorbate peroxidase from tomato (*LetAPX*) was expressed in Arabidopsis and conferred resistance to cold (4 °C for up to 24 h) [115]. Genetically modified tomato cultivars expressing the *A. thaliana Fe-SOD* gene promoted the increased performance and stability of the photosynthetic apparatus under UV stress [58].

#### 3.2.11. Ion Transport Proteins

Cation and anion transporters comprise a large class of transmembrane proteins vital for any organism, serving the purpose of moving ions and other small molecules within and between cells. These proteins are fundamental for ion homeostasis and are involved in salt stress resistance because they participate in sodium and chloride uptake, translocation, and cellular compartmentalization [116]. Numerous investigations have reported that genes like *HAL1* and *HAL5*, encoding ion transport proteins in *S. cerevisiae*, when delivered to tomato using recombinant DNA technology, increased the tolerance of toward salinity [117]. Similarly, the *A. thaliana AtNHX1* gene inserted in the tomato genome resulted in improved salinity tolerance [118]. In both cases, a positive effect was associated with an improved K/Na ratio under saline conditions. The importance of K homeostasis in the tolerance to NaCl stress was also demonstrated by overexpressing the endosomal LeNHX2 ion transporter [70,113].

## 4. Genome Editing for Ameliorating Abiotic Stresses in Tomato

New research tools and techniques are now available for creating abiotic stress-tolerant crop cultivars. The development of resistance to abiotic stress in crops through conventional breeding methods (e.g., crossing between two contrasting parental plants) has some drawbacks, such as the complex genetic base of the phenotypic features related to abiotic stress adaptation, and significant achievements have not been obtained [119,120]. Genetic engineering approaches based on the insertion of a transgene are restricted in certain areas of the world. Thus, to supply to the world’s rising population, there is great hope in genome editing tools, which are expected to revolutionize the world’s agriculture [121]. In tomato, genome editing has been performed for different genes, and resistance to abiotic stress has been obtained. For instance, the alteration of HyPRP1 (hybrid proline-rich protein 1) using CRISPR/CAS9 has resulted in salinity stress tolerance in mutagenized tomato lines [122]. Similarly, cold stress tolerance in tomatoes was obtained by mutation through CRISPR/CAS9 in the C-repeat binding factor 1 (CBF1) gene of tomato. The tomatoes with the cbf1 mutation were more vulnerable to stress and had increased electrolyte leakage [123]. To establish its function in tomato drought tolerance, the CRISPR/Cas9 method was recently utilized to create mutated strains for the tomato gene NPR1 (non-expresser of pathogenesis-related gene 1) [124]. S1NPR1 is essential for managing drought stress, and a variety of SlNPR1 variations may be created through genome editing to give tomato and other crops broad-spectrum drought tolerance.

## 5. Conclusions

Abiotic stress negatively influences the growth and yield of crops by disrupting their morpho-physio-biochemical and molecular activities. However, plants have evolved multiple counter-mechanisms. Advances in rDNA technology have made increasing the resistance to various abiotic stresses possible [77]. These recombinant DNA technologies are highly time-efficient compared to classic breeding and widely applicable to multiple plant species [38]. Despite the scientific and technological efficacy of biotechnological approaches, briefly presented in this review, there will always be constraints that can hamper the widespread diffusion of biotech tomatoes with abiotic stress tolerance. In different countries, this germplasm is subjected to a series of strict safety regulations, even when compared to genotypes derived from random mutagenesis programs. Currently, there is a strong debate on the possible legal distinction between plants obtained through classic genetic transformation (i.e., based on the transgene insertion) and plants developed using gene-editing approaches, such as CRISPR-CAS9. A strong argument that limits this distinction is that, in the EU (where GMOs are extensively regulated) any “organism in which the genetic material (DNA) has been altered in a way that does not occur naturally by mating or natural recombination”—and coherently, also plants obtained by site-directed mutagenesis—“are subject to the obligations laid down by the GMO Directive” (European Court of Justice ruling of case C-528/16, 2018; https://curia.europa.eu, accessed on the 1 December 2022). Transgenic plants are the most common GMOs but not the only possible GMOs. In the EU, GMOs can be eventually marketed only after a scientific assessment of the risks to health and the environment. Under the current legal framework, any molecular biology technique that induces DNA variations with a mechanism that does not occur in nature will generate a GMO. We would like to conclude that, rather than focusing on the technique that alters the DNA or the source of the genetic material, a less stringent legal framework and a less complex and onerous authorization procedure can be perhaps granted for organisms modified using BBA because this approach can better achieve the development of modified varieties within the functional range potentially obtainable by conventional methods or classic mutagenesis.

## Figures and Tables

**Table 1:** Investigations focused on improving the efficiency of tomato genetic transformation.

*S. lycopersicum* Cultivar	Transformation Method	Type of Explant	Transformation Frequency (TF)	References
Micro-Tom	Indirect	Embryonic part of the seedling	11%	[41]
NA	Indirect	Fruits	54 to 68.0%	[44]
Micro-Tom	Indirect	Cotyledons (embryonic part)	5.1%	[45]
Hezuo 908	Indirect	Hypocotyls and embryonic part	40%	[40]
Roma and Rio Grande	Indirect	Hypocotyls and leaf disks	24% and 8%, respectively	[4]
Momotaro, UC-97, and Edkawi	Indirect	Hypocotyls	54 to 67%	[46]
Castle Rock	Direct	Hypocotyls and part of cotyledons	26.5%	[47]
Cambell-28	Indirect	Cotyledons	21.5%	[48]
Pusa Ruby, Sioux, and Arka Vikas	Indirect	Cotyledons	41.4%, 22%, and 41%, respectively	[49]
Hezuo 908	Indirect	Embryonic part and Hypocotyl	40%	[40]
Shalimar	Indirect	Shoot and Leaf	NA	[50]
MicroTom	Indirect	Leaf	19.1%	[51]
NA	Indirect	Hypocotyls	33 to 59%	[41]
Pusa Ruby and DT-93	Indirect	Cotyledons	higher than 37%	[52]
Summer	Indirect	Hypocotyls and cotyledons	7%	[53]

Footnote. NA: not available.

**Table 2:** A selection of genes that have been utilized to improve tomato cultivars to withstand abiotic stress.

Gene/Origin	Function	Expression	Results	References
Fe-SOD/*A. thaliana*	Lessens the oxidative stress	Upregulation	Increased ability to withstand oxidative stress and improve stability of photosynthetic equipment	[58]
SIERF3b & SIERF5	Regulates transcription for stress conditions	Overexpression	Enhanced tolerance to abiotic stress and resistance to biotic stress	[59]
FAD3/rape FAD7/potato	Regulates the fatty acid unsaturation of membrane lipids	Upregulation	Boosted cold resistance; an increase in the 18:3/18:2 ratio in leaves and fruits	[60]
SlSAM1/tomato	Promotes the conversion of ATP plus methionine to S-adenosylmethionine, which is necessary for the production of ethylene and PAs	Upregulation	Improved resistance to saline-alkali stress	[61]
SIGGP (LIKE)/tomato	Transcription factor	Expression	Enhanced tolerance to abiotic and biotic stress	[62]
SIBZIP1/tomato	TF, Defense protein	Downregulation	Regulated ABA-mediated pathway to enhance drought tolerance	[63]
*SLWRKY*	TF, transcriptional regulation	Overexpression	Regulated biotic stress	[64]
RcGPX5/*Salvimiltiorrhiza*	Gluthatione biosynthesis	Overexpression	Tolerance to H_2_O_2_, drought and oxidative stress	[65]
CBF1/*A. thaliana*	TF and regulates transcription	Transcription/ regulation	Enhanced cold tolerance	[66]
MdVHA-B/apple	Maintains the homeostasis of ion	Upregulation	Improved drought tolerance	[67]
MdSOS2L1/apple	Signal-inducing proteins; influence on ion-driving transport mechanisms	Excessive expression	Increased salt tolerance	[68]
TERF1/sugarcane	Transcription factor for ethylene response; assimilates ethylene and osmotic stress pathways	Excessive expression	Increased tolerance to drought stress, osmotic stress caused by salt	[69]
LeNHX2/tomato	Transport of ions	Expression in excess	Salt tolerance increase	[70]
CWIN (Lin7)/tomato	Takes part in mechanisms related to temperature stress	Expresses at normal level	Improved heat resistance in tomato flowers	[71]
mt1D/*E. coli*	Biosynthesis of mannitol	Upregulation	Increased tolerance to drought, cold, and salinity	[72]
LeFAD7/tomato	Role in fatty acid transcription	Antisense regulation	Improved high-temperature tolerance; trienoic fatty acids reduced	[73]
TPSP (TPS/TPP fusion gene)/*E. coli*	Biosynthesis of Trehalose	Upregulation	Salt and drought resistance improved	[74]
SlICE1/tomato	Transcription regulation	Overexpression	Improved tolerance to cold	[75]
katE/tomato	Oxidative stress (catalase)	Overexpression	Upgraded resistance to photo-oxidative stress as a result of drought and Fungal stress	[76]
tas14/tomato	Accumulates chaperone-like proteins more effectively	upregulation	Enhanced tolerance to drought and salinity without any growth aberrations	[77]
Glycine betaine	Stress savior	Supplements	Chilling tolerance increased	[78]
ZAT12/*B. carinata*	Transcriptional regulation	Upregulation	Boosted resistance toward drought	[79]
CaKR1/pepper	Impact on defense machinery	Expression in excess	Improved salt tolerance as well as oxidative stress	[80]
ZAT12/*B. carinata*	Transcription of C2H2 zinc finger protein	Upregulation	Enhanced tolerance to heat	[81]
PtADC/*P. trifoliata*	Involved in PAs synthesis	Upregulation	Increased tolerance to water stress	[82]
LeFAD3/tomato	Transcription of fatty acid and lipids unsaturation	Transcribed in excess	Augmented tolerance to salt stress	[83]
TaNHX2/wheat	Transport of ions; equal-ion management	Upregulation	Boosted resistance to salt stress	[84]
LeHSP21.5/tomato	Heat shock protein	Overexpression	Combat with tunicamycin-induced stress	[85]
MdPIP1; 3/Apple	Aquaporin protein and used to increase fruit size	Expression	Increased drought tolerance	[86]
OSMOTIN gene/*N. Tobacum*	Has a higher level of proline	Constitutively expression	Tolerance to salt stress	[87]
CodA/tomato	Organic osmolyte	Expression	Enhanced chilling stress	[88]

## Data Availability

Not applicable.
